# Models for predicting risk of endometrial cancer: a systematic review

**DOI:** 10.1186/s41512-024-00178-0

**Published:** 2025-02-04

**Authors:** Bea Harris Forder, Anastasia Ardasheva, Karyna Atha, Hannah Nentwich, Roxanna Abhari, Christiana Kartsonaki

**Affiliations:** 1 Medical Sciences Division, University of Oxford, Oxford, UK; 2 Clinical Trials Service Unit and Epidemiological Studies Unit (CTSU), Nuffield, Department of Population Health (NDPH), Big Data Institute Building , University of Oxford, Old Road Campus, Oxford, OX3 7LF UK

**Keywords:** Endometrial cancer, Risk prediction, Early detection

## Abstract

**Background:**

Endometrial cancer (EC) is the most prevalent gynaecological cancer in the UK with a rising incidence. Various models exist to predict the risk of developing EC, for different settings and prediction timeframes. This systematic review aims to provide a summary of models and assess their characteristics and performance.

**Methods:**

A systematic search of the MEDLINE and Embase (OVID) databases was used to identify risk prediction models related to EC and studies validating these models. Papers relating to predicting the risk of a future diagnosis of EC were selected for inclusion. Study characteristics, variables included in the model, methods used, and model performance, were extracted. The Prediction model Risk-of-Bias Assessment Tool was used to assess model quality.

**Results:**

Twenty studies describing 19 models were included. Ten were designed for the general population and nine for high-risk populations. Three models were developed for premenopausal women and two for postmenopausal women. Logistic regression was the most used development method. Three models, all in the general population, had a low risk of bias and all models had high applicability. Most models had moderate (area under the receiver operating characteristic curve (AUC) 0.60–0.80) or high predictive ability (AUC > 0.80) with AUCs ranging from 0.56 to 0.92. Calibration was assessed for five models. Two of these, the Hippisley-Cox and Coupland QCancer models, had high predictive ability and were well calibrated; these models also received a low risk of bias rating.

**Conclusions:**

Several models of moderate-high predictive ability exist for predicting the risk of EC, but study quality varies, with most models at high risk of bias. External validation of well-performing models in large, diverse cohorts is needed to assess their utility.

**Registration:**

The protocol for this review is available on PROSPERO (CRD42022303085).

**Supplementary Information:**

The online version contains supplementary material available at 10.1186/s41512-024-00178-0.

## Introduction

Endometrial cancer (EC) is the most common gynaecological cancer in the UK and the eighth most common cause of cancer death in UK females, causing around 2500 deaths annually [1]. Globally, in 2019, there were 435,041 new cases of EC [2]. The rate of EC is increasing in many countries and across all age groups [3]; indeed, it is estimated that EC cases may reach 600,000 in 2044 [2]. This rise in cases is likely explained by increases in the prevalence of obesity and other factors that increase the risk of developing EC [4].

EC typically presents early due to the malignant process within the endometrium causing abnormal uterine bleeding. Whilst postmenopausal bleeding (PMB) is sensitive for EC (detecting around 90% of postmenopausal cases) [5], it is not specific; the prevalence of EC in women with PMB is only around 9% [6]. Other symptoms may present in the later stages. Reflecting its often early presentation, EC typically has a good prognosis; however, high-grade and serous subtypes have a much poorer prognosis [7, 8].

The presentation of EC in premenopausal women is harder to discern, due to the physiological circulation of reproductive hormones. In this population, it may cause menorrhagia, intermenstrual bleeding, and changes in discharge, all of which could be explained by benign pathologies.

EC diagnosis may include a combination of transvaginal ultrasound scan, hysteroscopy, and endometrial sampling in a single hospital visit; however, these invasive procedures can be associated with severe pain for some women and moderate pain for most [9]. The non-specific nature of the typical presenting symptoms results in many women with benign conditions undergoing invasive testing [10].

There are a number of established risk factors for EC; the most important of which is adiposity which leads to conversion of androgens by aromatase in adipocytes to oestrogen [11], which subsequently exerts proliferative effects on the endometrium. Insulin activates the same pro-proliferative signalling pathways as oestrogen; hence, conditions in which insulin is dysregulated, such as diabetes mellitus (DM) and polycystic ovarian syndrome (PCOS), also carry the risk for EC [12]. Other oestrogenic risk factors include oestrogen-only HRT [13], tamoxifen [14], early menarche [15], and late menopause [16], whilst parity is a protective factor [17]. Certain genetic syndromes are also associated with EC. Lynch syndrome is caused by germline mutations in DNA mismatch repair genes MSH2, MSH6, MLH1, PMS2, or EPCAM, with a risk of EC of up to 54% by the age of 70 [18], as well as an elevated risk of colorectal and ovarian cancer. Lynch syndrome is rare and causes approximately 3% of EC cases [19]. Cowden syndrome is very rare, with an estimated incidence of around 1 in 200,000 [20], mainly caused by pathogenic germline PTEN mutations, and carries a high risk of EC, breast, thyroid, kidney cancers, and melanoma. The lifetime risk of EC in Cowden syndrome has been estimated to be 28% [21].

Risk prediction models can be used to estimate an individual’s probability of developing cancer. They can be useful when clinicians need to estimate a person’s risk when considering how urgently a patient should be further assessed and considering a differential diagnosis. Models could also prove to be a good adjunct to streamline the decision-making regarding which women end up undergoing biopsy, allowing more informed clinical decisions to be made prior to progressing to investigation. Moreover, models could identify women in whom lifestyle adjustments should be encouraged, to reduce the risk of EC development. A need for such models has been highlighted: a 2016 gap analysis [22] identified a key area in EC research is the development of risk-scoring systems for EC and a stratification system for women with abnormal vaginal bleeding to determine who needs referral for secondary investigations.

Several risk prediction models for EC have been developed, but as of yet, no summary of available models predicting future EC risk exists. This systematic review summarises risk prediction models for EC, the variables they include, and their performance, with the aim of identifying models with strong performance, and those which can be used in practice settings with different levels of resources. A summary of these models would be useful in external validation and would allow them to be further adapted to improve their predictive ability. This carries the potential for utilisation in predicting future EC in symptomatic and asymptomatic women.

## Methods

The MEDLINE and Embase (OVID) databases were searched from inception to the 29th of January 2022, identifying articles on ovarian, endometrial/uterine, cervical, vaginal, and vulvar cancers, the risk prediction models related to these cancers and validation studies testing these models (see PROSPERO CRD42022303085). Here we only consider endometrial cancer models, with other gynaecological cancers considered in other papers. The search (Appendix S1) was subsequently re-run on the 20th of January 2023 and the 3rd of December 2023 in order to identify studies published between the search dates. To be included, papers had to be written in the English language. Conference papers and abstracts, as well as studies assessing performance of diagnostic tools and techniques, were excluded. We did, however, include studies which aimed to predict short-term risk of endometrial cancer, for example, diagnosis over the next 1–2 years. Prior to the final analysis, the references of included articles and reviews were searched for inclusion.

Title and abstract screening and full-text screening were performed independently by two reviewers (AA/BF, CK) using Rayyan, after excluding duplicates, with disagreements settled by discussion. Data extraction was performed independently by two reviewers (BF, KA/HN) and extracted data were then consolidated. The extracted data included study information, study design, the size and type of population, the variables included in the risk models, and measures of model performance including the area under the receiver operating characteristic curve (AUC) as a measure of discrimination, sensitivity, specificity, positive predictive value (PPV), negative predictive value (NPV), and measures of calibration such as the Brier score, Hosmer–Lemeshow statistic, Goodness of Fit χ^2^ statistic, and the ratio of observed to expected cases. In cases where models provided calibration plots but not statistics, the original authors’ judgement of the calibration plots was extracted.

The Prediction Model Risk Of Bias Assessment Tool (PROBAST) [23] was used to assess the risk of bias and applicability of models. This tool assesses four domains for potential risk of bias: participants, predictors, outcome, and analysis. In general, this tool assigns a low risk of bias rating to studies which use a robust study design, clearly define outcomes, predictors, and time frame and validate their models, and provide acceptable measures of discrimination and calibration. Regarding the latter, whilst Hosmer–Lemeshow statistics were extracted; they were not deemed an acceptable measure of calibration, and hence, papers reporting this as their sole measure of calibration could not receive a ‘low’ risk of bias rating in the analysis domain. Three domains of applicability are assessed: participants, predictors, and outcome. A high applicability is assigned to papers whose study population, variables incorporated, and outcomes are in line with the scope of the review question. For each domain, studies were rated as having a ‘high’, ‘low’, or ‘unclear’ risk of bias or a ‘high’, ‘low’, or ‘unclear’ concern regarding applicability. The tool was used independently by two authors (BF, KA/HN), with discrepancies in these domains resolved by discussion.

The PRISMA reporting guidelines were followed throughout the process of reporting this systematic review.

## Results

### Model characteristics

The initial search and two subsequent updates of this, for risk prediction models spanning ovarian, endometrial/uterine, and cervical cancers, identified 6494 titles after the removal of duplicates. One hundred and twenty-three studies were selected for full-text screening and were subsequently split by cancer type; this paper only discusses those including EC, with 20 papers [24–43] on EC selected for inclusion (eFigure 1). Baak et al. [41] is a validation study of a model proposed in one of their earlier papers [43]; hence, the total number of models assessed was 19. Table 1 provides a summary of the included studies including descriptions, locations and population types, and study type. Six of the studies were from the UK [29, 31, 33, 37, 38, 40], with others from the US [25, 28, 39], Norway [26, 41], Taiwan [30], India [32], Italy [34], the Netherlands [41, 43], and Sweden [42]. One of the studies was based both in the Netherlands and Norway [41]. Some utilised data from large multi-centre cohort studies: two used data from the European Prospective Investigation into Cancer and Nutrition (EPIC) cohort [35, 36] comprised of ten European countries; one was part of the FORECEE (4C) programme [24] which is based in five European countries and another utilised data from the Epidemiology for Endometrial Cancer Consortium (E2C2) [28]. Ten models were developed in the general population [24, 28, 29, 31, 33, 35–39] and nine in a high-risk population [25–27, 30, 32, 34, 41–43]. High-risk individuals were defined as having abnormal uterine bleeding [25, 27, 32, 34, 40], endometrial hyperplasia [26, 30, 41, 43], or on the basis of having received a referral to gynaecological oncology [42]. Fourteen models were developed for use in all women [24, 26, 27, 29–31, 33, 35–39, 42, 43], with three models developed for premenopausal women [30, 34, 37, 38], and two for postmenopausal women [28, 40]. The number of EC cases used in model development varied from 3 [34] to 6949 [37]. Four models explicitly stated a time frame for risk prediction [30, 36–38]. Although studies did not always make the time frame over which they predict future risk clear, reported outcomes ranged from a 2-year risk [38] to a lifetime risk of developing EC. Three models, Baak et al. [43], Burbos et al. [40], and Hippisley-Cox and Coupland [38] predicted risk over a shorter time (< 4 years), whilst other models predicted risk over longer periods.

**Table 1:** Summary of included studies

Author(s), year	Model name (if applicable)	Model description	Location	Study type
**General population**				
Barrett et al. 2023 [24]	WID-EC	Classifier index based on ridge and lasso regression	Europe (FORECEE programme)^a^	Development and validation
Shi et al. 2023 [28]		Logistic regression	US	Development and validation
Bafligil et al. 2022 [29]		Polygenic risk score	UK	Development and validation
Hutt et al. 2021 [31]		Neural network	UK	Development and validation
Choi et al. 2020 [33]		Cox regression including polygenic risk scores	UK (UK Biobank)	Development only
Fortner et al. 2017 [35]		Conditional logistic regression	Europe (EPIC Cohort)^a^	Development and validation
Hüsing et al. 2016 [36]		Cox regression	Europe (EPIC Cohort)^a^	Development and validation
Hippisley-Cox and Coupland 2015 [37]	QCancer®	10-year risk algorithms based on Cox proportional hazard models	UK	Development and validation
Hippisley-Cox and Coupland 2013 [38]	QCancer®	Multinomial logistic regression-based	UK	Development and validation
Pfeiffer et al. 2013 [39]		Cox regression-generated absolute risk scores	US	Development and validation
**High-risk population**				
Beavis et al. 2023 [25]		Multivariate Poisson regression	US	Development only
Rewcastle et al. 2023 [26]	ENDOAPP	Logistic regression	Norway	Development only
Ruan et al. 2023 [27]		Multivariate logistic regression	China	Development and validation
Lin et al. 2022 [30]		Measures the levels of different miRNAs, used solely and in combination with PTEN loss	Taiwan	Development and validation
Bagepalli Srinivas et al. 2020 [32]	PAD30	Score based on variables identified by multivariate regression analysis	India	Development and validation
Giannella et al. 2019 [34]		Multivariate logistic regression	Italy	Development only
Burbos et al. 2010 [40]	Norwich DEFAB risk assessment tool	Logistic regression	UK	Development only
Baak et al. 2001 [41]	D-Score	External validation of their previously described D-score	Netherlands, Norway	Validation only
Dahlgren et al. 1989 [42]		Linear model	Sweden	Development only
Baak et al. 1988 [43]	D-Score	Linear model	Netherlands	Development only

^a^Details provided in eTable 1

### Model development

Logistic regression, with various regularisation techniques, was the most commonly used method [24, 26–29, 32, 34, 35, 38, 40], followed by Cox regression [33, 36, 37, 39]. One study, Hutt et al. [31], used a neural network model incorporating medical and lifestyle factors.

**Fig. 1:**
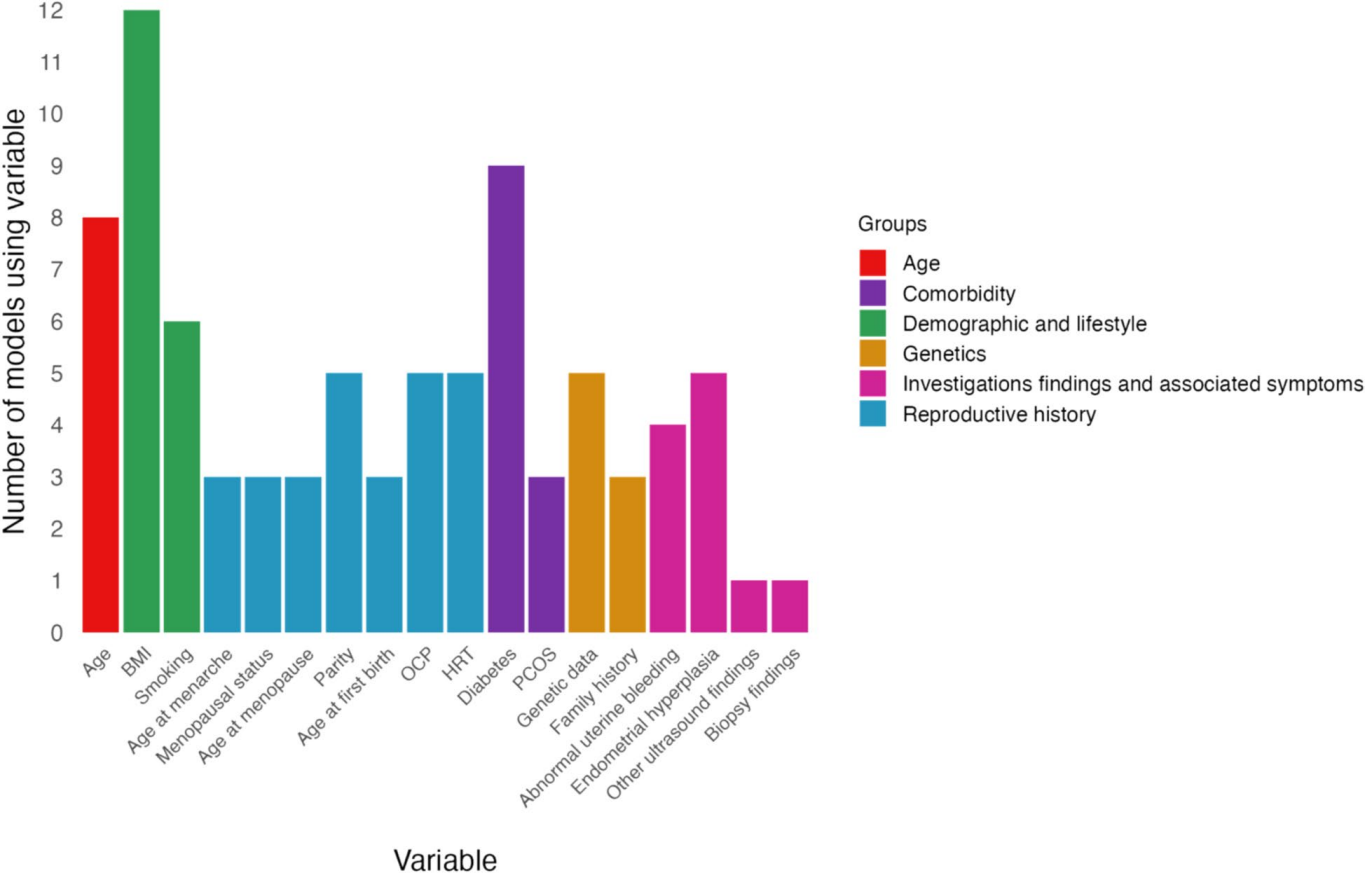
Frequency of variable use

The variables included in the risk prediction models were grouped into the following categories: age, demographic and lifestyle, reproductive history, comorbidities, genetics, investigation findings and associated symptoms, and others. Tables 2 and 3 provide a summary of the variables included in the different models in general and high-risk populations, with Fig. 1 showing the number of models using each variable. Demographic and lifestyle information was used most commonly, with 13 models including either BMI, smoking, or both [25, 27, 28, 31, 32, 34–40, 42]. The least utilised category was reproductive history, which was used by six models. No model included variables from all six categories. Four models used variables from five of the categories [27, 30, 37, 38]. Three models included variables from four categories [32, 40, 42], each including age, demographic and lifestyle, and investigation findings and associated symptoms. Three models included variables from three categories: Beavis et al. [25] from age, demographic and lifestyle, and comorbidity; Hutt et al. [31] from demographic and lifestyle, reproductive history, and comorbidity; and Giannella et al. [34] from demographic and lifestyle, comorbidity, and investigation findings and associated symptoms. Three of the papers included variables from two categories: [35, 36, 39] demographic and lifestyle, and reproductive history. Six models utilised variables from only a single category [24, 26, 29, 30, 33, 43], most commonly genomic-related information (for example SNVs, CpG methylation, and miRNA expression); [24, 29, 30, 33] two models, Baak et al. [43] and Rewcastle et al. [26] were developed based on histological findings of endometrial biopsy [26, 43].

**Table 2:** Summary of variables included in models in the general population

		Barrett et al. 2023 [24]	Shi et al. 2023 [28]	Bafligil et al. 2022 [29]	Hutt et al. 2021 [31]	Choi et al. 2020 [33]	Fortner et al. 2017 [35]	Hüsing et al. 2016 [36]	Hippisley-Cox and Coupland 2015 [37]	Hippisley-Cox and Coupland 2013 [38]	Pfeiffer et al. 2013 [39]
**Age**			X						X	X	
**Demographic and lifestyle**	BMI		X		X		X	X	X	X	X
	Smoking		X				X	X	X	X	X
**Reproductive history**	Age at menarche		X				X	X			
	Menopausal status						X	X			X
	Age at menopause						X	X			X
	Parity		X		X		X	X			X
	Age of first live birth		X				X	X			
	OCP use		X		X		X	X			X
	HRT use		X		X		X	X			X
**Comorbidities**	Diabetes		X		X				X	X	
	PCOS				X				X		
**Genetics**	Genetic data	CpG methylation of endometrial tissue	SNV data	SNV data		SNV data					
	Family history								X	X	
**Investigation findings and associated symptoms**	Abnormal uterine bleeding					X				IMB, PMB	
	Endometrial hyperplasia								X	X	
	Other USS findings										
	Biopsy findings (histology)										
**Others**			Education status, hypertension		IUD		Serum biomarkers		Endometrial polyps, manic depression, schizophrenia, previous breast and bowel cancer, family history of other gynaecological cancers	Deprivation score, COPD, endometrial polyp, anaemia, VTE, abdominal pain, haematuria

**Table 3:** Summary of variables included in models in high-risk populations

		Beavis et al. 2023 [25]	Rewcastle et al. 2023 [26]	Ruan et al. 2023 [27]	Lin et al. 2022 [30]	Bagepalli Srinivas et al. 2020 [32]	Giannella et al. 2019 [34]	Burbos et al. 2010 [40]	Dahlgren et al. 1989 [42]	Baak et al. 1988 [43]
**Age**		X	X	X		X		X	X	
**Demographic and lifestyle**	BMI	X		X		X	X	X	X	
	Smoking								X	
**Reproductive history**	Age at menarche									
	Menopausal status									
	Age at menopause									
	Parity								X	
	Age of first live birth									
	OCP use									
	HRT use									
**Comorbidities**	Diabetes	X		X		X	X	X		
	PCOS	X								
**Genetics**	Genetic data				miRNA expression in endometrial tissue					
	Family history			X						
**Investigation findings and associated symptoms**	Abnormal uterine bleeding					X		X	X	
	Endometrial hyperplasia			X			X	X		
	Other USS findings			Blood flow RI						
	Biopsy findings (histology)		X							X
**Others**		Ethnicity							Hirsutism	

*Abbreviations:* *BMI* body mass index, *COPD* chronic obstructive pulmonary disease, *HRT* hormone replacement therapy, *IMB* intermenstrual bleeding, *IUD* intrauterine device, *OCP* oral contraceptive pill, *PCOS* polycystic ovarian syndrome, *PMB* postmenstrual bleeding, *RI* resistive index, *SNV* single nucleotide variant, *USS* ultrasound scan, *VTE* venous thromboembolism

### Risk of bias and applicability in the general population

The risk of bias and applicability of the risk prediction models were assessed using the PROBAST tool (Table 4). Of the ten models designed for use in the general population, three models were deemed to be at low risk of bias. These were both the Hippisley-Cox and Coupland QCancer models [37, 38] and Hüsing et al. [36]. These models were developed in large populations with appropriate study design, and the studies included either internal or external validation and presented relevant measures of model performance, that is discrimination and calibration. Barrett et al. [24], Shi et al. [28], Bafligil et al. [29], and Hutt et al. [31] all received a rating of high risk of bias in the participants domain for being based on a case–control study design. Hutt et al. [31] and Choi et al. [33] received a high risk of bias rating in the analysis domain. In the case of Hutt et al. this was due to inappropriate treatment of continuous variables, whilst Choi et al. were penalised for not including validation or calibration. Fortner et al. [35] received an unclear risk of bias rating, as whilst they included 1000-fold bootstrapped internal validation they did not include a measure of calibration. All models except Shi et al. [28], the QCancer models [37, 38], and Hüsing et al. [36] received an unclear risk of bias rating for the outcome domain due to not explicitly stating a time frame of risk prediction [39].

**Table 4:** PROBAST assessment^a^ for included studies

Author(s), year	ROB				Applicability			Overall	
	**Participants**	**Predictors**	**Outcome**	**Analysis**	**Participants**	**Predictors**	**Outcome**	**ROB**	**Applicability**
**General population**									
Barrett et al. 2023 [24]	-	+	?	?	+	+	+	-	+
Shi et al. 2023 [28]	-	+	+	+	+	+	+	+	+
Bafligil et al. 2022 [29]	-	+	?	?	+	+	+	-	+
Hutt et al. 2021 [31]	-	+	?	-	+	+	+	-	+
Choi et al. 2020 [33]	+	+	?	-	+	+	+	-	+
Fortner et al. 2017 [35]	+	+	?	?	+	+	+	?	+
Hüsing et al. 2016 [36]	+	+	+	+	+	+	+	+	+
Hippisley-Cox and Coupland 2015 [37]	+	+	+	+	+	+	+	+	+
Hippisley-Cox and Coupland 2013 [38]	+	+	+	+	+	+	+	+	+
Pfeiffer et al. 2013 [39]	+	+	?	+	+	+	+	+	+
**High-risk population**									
Beavis et al. 2023 [25]	-	+	?	-	+	+	+	-	+
Rewcastle et al. 2023 [26]	+	+	-	-	+	+	+	-	+
Ruan et al. 2023 [27]	-	-	+	-	+	+	+	-	+
Lin et al. 2013 [30]	-	+	?	-	+	+	+	-	+
Bagepalli Srinivas et al. 2020 [32]	-	-	?	-	+	+	+	-	+
Choi et al. 2020 [33]	+	+	?	-	+	+	+	-	+
Giannella et al. 2019 [34]	-	-	?	-	+	+	+	-	+
Burbos et al. 2010 [40]	+	+	?	-	+	+	+	-	+
Baak et al. 2001 [41]	-	+	-	-	+	+	+	-	+
Dahlgren et al. 1989 [42]	?	+	?	-	+	+	+	-	+
Baak et al. 1988 [43]	?	+	?	-	+	+	+	-	+

^a^*PROBAST* prediction model risk of bias assessment tool, *ROB* risk of bias

The positive symbol “ + ” indicates low-risk ROB/concern regarding applicability; the negative symbol “ − ” indicates high ROB/concern regarding applicability; the question mark “?” indicates unclear ROB/concern regarding applicability

All models in the general population received a rating of low concern for applicability as the participants, predictors, and outcomes of each of them were felt to be within the scope of the review question.

### Risk of bias and applicability in the high-risk population

Of the nine models developed for high-risk populations, none received a rating of a low risk of bias [25–27, 30, 32, 34, 41–43]. All received a rating of high risk of bias in the analysis domain, due to inappropriate handling of continuous or categorical predictors, failing to include both validation and calibration, or having events (in this case number of EC cases) per variable of less than ten. Giannella et al. [34] developed their model with the lowest number of cases, with only three EC cases. Ruan et al. [27], Lin et al. [30], Bagepalli Srinivas et al. [32], Giannella et al. [34], and Baak et al. [43] all also received a rating of high risk of bias for participants. Two of the models were developed for use in premenopausal women: Bagepalli Srinivas et al. [32] and Giannella et al. [34], both had included endometrial thickness as a variable and were rated as having a high risk of bias in the predictors’ domain due to the fluctuations in endometrial thickness throughout the menstrual cycle making this an unreliable predictor. Ruan et al. [27] received a rating of unclear risk of bias in the predictors’ domain due to ambiguity in how and when their predictors were assessed. No model received a rating of low risk of bias in the outcome domain, with most receiving an unclear rating for not stating a time frame of risk prediction, with the exception of Rewcastle et al. [26] and Baak et al. [41] who received high risk of bias rating.

Similarly to the models in the general population, all models in the high-risk populations received a rating of low concern regarding applicability, as they were deemed to have participants, predictors, and outcomes that fit with the review question.

### Performance of models developed in the general population

The predictive ability of all included models is summarised in Table 5. Ten models were developed for use in the general population [24, 28, 29, 31, 33, 35–39]. Of these, eight provided measures of discrimination in the form of an AUC [24, 31, 33, 35–39]. Three models had a high AUC (> 0.80). Barrett et al. [24] reported an AUC of 0.92 (95% CI 0.88–0.97) in internal validation in a retrospective case–control sample, whilst prospective external validation yielded an AUC of 0.82 (95% CI 0.74–0.89). The models in Hippisley-Cox and Coupland’s [37, 38] papers performed similarly, with AUCs of 0.83 (95% CI 0.82–0.84) and 0.910 (95% CI 0.90–0.93) respectively in internal validation. Shi et al. [28], Fortner et al. [35], Hüsing et al. [36], and Pfeiffer et al. [39] reported moderate AUCs (ranging between 0.60 and 0.80). Shi et al. [28] provide AUCs ranging from 0.64 to 0.69 for their epidemiological model and 0.61 to 0.67 for their epidemiological and genetic model. Fortner et al. [35] described two models, with AUCs of 0.69 95% CI 0.64–0.73) for model 1 and 0.68 (95% CI 0.64–0.72) for model 2. Hüsing et al. [36] reported an AUC of 0.77 (95% CI 0.68–0.85) and Pfeiffer et al. [39] included an AUC of 0.68 (95% CI 0.66–0.70). The other two models designed for use in the general population that assessed model discrimination reported AUCs that suggest poor predictive ability (< 0.60). These were Bafligil et al. [29] who reported AUCs ranging between 0.53 and 0.56 in external validation, and Choi et al. [33] whose model had an AUC of 0.56 (95% CI 0.54–0.58).

**Table 5:** Performance of the endometrial cancer risk prediction models

Author(s), year	Development population size (*n*)	Development population age (range and/or mean (SD))	Validation population size (*n*)	Validation population age (range and/or mean (SD))	Model performance		
					**Discrimination (AUC (95% CI))**	**Calibration**	**Other metrics**
**General population**							
Barrett et al. 2023 [24]	1086 (217 EC cases)		*Internal validation* 289 (64 EC cases) *Prospective external validation* 150 (54 EC cases)		*Internal validation* 0.920 [0.880–0.970] *Prospective external validation* 0.82 [0.74–0.89]		**WID-EC ≥ 0.14:** Sensitivity 86% Specificity 95% *Internal validation* **WID-EC ≥ 0.14:** Sensitivity 86% Specificity 90% *Prospective external validation* **WID-EC ≥ 0.14:** Sensitivity 52% Specificity 98%
Shi et al. 2023 [28]	15,727 (6665 EC cases)	63.1 (EC cases) 62.8 (control)	NHS 68,150 NHS II 56,076 PLCO 30,102		*External validation* Epidemiologic model NHS 0.65 [0.63–0.67] NHS II 0.69 [0.66–0.72] PLCO 0.64 [0.61–0.66] Epidemiologic and genetic model NHS 0.61 [0.57–0.66] PLCO 0.67 [0.64–0.69]	Epidemiologic model NHS E/O ratio 0.55 [0.51–0.59] Hosmer–Lemeshow statistic 151.7 (*p* < 0.001) Goodness of fit *χ*^2^ 33.0 (*p* < 0.001) NHS II E/O ratio 1.09 [0.98–1.22] Hosmer–Lemeshow statistic 14.2 (*p* = 0.165) Goodness of fit *χ*^2^ 15.3 (*p* = 0.084) PLCO E/O ratio 1.04 (0.95–1.13) Hosmer–Lemeshow statistic 21.3 (*p* = 0.019) Goodness of fit *χ*^2^ 20 (*p* = 0.018) Epidemiologic and genetic model PLCO E/O ratio 0.94 (0.85–1.03) Hosmer–Lemeshow statistic 20.5 (*p* = 0.025) Goodness of fit *χ*^2^ 27.6 (*p* = 0.001)	
Bafligil et al. 2022 [29]	1752 (555 EC cases)	62.23 (EC cases) 59 (control)	118,636 (1676 EC cases)	61 (EC cases) 55 (control)	PRS19 0.59 [0.56–0.61] PRS24 0.55 [0.52–0.58] PRS72 0.57 [0.54–0.60] *External validation* AUC: PRS19 0.56 [0.54–0.57] PRS24 0.53 [0.52–0.54] PRS72 0.54 [0.52–0.55]		
Hutt et al. 2021 [31]			1200				*External validation* Accuracy 98.6% Sensitivity 75% Specificity 98.78%
Choi et al. 2020 [33]	214,435 (629 EC cases)				0.56 [0.54–0.58]		
Fortner et al. 2017 [35]	716 (247 EC cases)	57	1000 boostrapped samples		Model 1 0.69 [0.64–0.73] Model 2 0.68 [0.64–0.72]		
Hüsing et al. 2016 [36]	208,811 (855 EC cases)	50			0.77 [0.68–0.85]	E/O ratio 0.99	
Hippisley-Cox and Coupland 2015 [37]	2,495,899 (6949 EC cases)	25–84 44.6 [15.9]	822,359	44.1 [15.9]	*Internal validation* 0.83 [0.82–0.84]	Plot of mean predicted risks and observed risks at 10 years by tenth of predicted risk Interpretation: ‘well calibrated’	*Internal validation* Sensitivity 42.6% Specificity 90.1%
Hippisley-Cox and Coupland 2013 [38]	1,240,864 (1015 EC cases)	25–89 50.3 [17.5]	667,603	50.1 [17.4]	*Internal validation* 0.91 [0.90–0.93]	Plot of mean predicted risks and observed risks at 10 years by tenth of predicted risk Interpretation: ‘well calibrated’	
Pfeiffer et al. 2013 [39]	146,679 (1559 EC cases)		37,241		0.68 [0.66–0.70]	E/O ratio 1.20 [1.11–1.29]	
**High-risk population**							
Beavis et al. 2023 [25]	3175						
Rewcastle et al. 2023 [26]	363	24–88 51			0.77 [0.66–0.79]		**XR scanner** Sensitivity 50% Specificity 92% PPV 0.38 NPV 0.95 **S60 scanner** Sensitivity 42% Specificity 96% PPV 0.52 NPV 0.94
Ruan et al. 2023 [27]	1369		468		0.84 [0.80–0.87] *External validation* 0.91 [0.88–0.94]	Plot of predicted and observed frequencies *Development* Mean absolute error 0.005 Interpretation ‘good’ *Validation* Mean absolute error 0.053 Interpretation ‘acceptable’	
Lin et al. 2022 [30]	119 (25 EC cases)				miR-30a-3p 0.62 [0.48–0.77] miR-141 0.75 [0.63–0.87] miR-200a 0.78 [0.66–0.91] miR-200b 0.70 [0.56–0.85]		**miR-30a-3p** Sensitivity 60% Specificity 67.4% **miR-141** Sensitivity 48% Specificity 96.6% **miR-200a** Sensitivity 60% Specificity 100% **miR-200b** Sensitivity 56% Specificity 89.9% **PTEN** Sensitivity 52% Specificity 100% **miR-30a-3p + PTEN** Sensitivity 50% Specificity 98.9% **miR-141 + PTEN** Sensitivity 50% Specificity 100% **miR-200a + PTEN** Sensitivity 76% Specificity 100% **miR-200b + PTEN** Sensitivity 60% Specificity 100%
Bagepalli Srinivas et al. 2020 [32]	236 (14 EC cases)		236 (14 EC cases), validated via split sampling		*Internal validation* 0.85 [0.75–0.93]		*Internal validation* **PAD30 ≥ 5** Sensitivity 85.7% Specificity 87.6% PPV 0.306 NPV 0.989
Giannella et al. 2019 [34]	240 (3 EC cases)				0.85 [0.80–0.90]		**ET > 11** Sensitivity 75% Specificity 90.79% PPV 0.300 NPV 0.986
Burbos et al. 2010 [40]	3,047 (149 EC cases)				Overall DEFAB 0.77 DEFAB ≥ 3 0.66 DEFAB ≥ 5 0.71		**DEFAB ≥ 3** Sensitivity 81.9% Specificity 67.8% PPV 0.0778 NPV 0.982 **DEFAB ≥ 5** Sensitivity 67.8% Specificity 74.1% PPV 0.119 NPV 0.978
Baak et al. 2001 [41]			132				*Internal validation of *[43] Accuracy 86% D ≤ 0 vs > 1: Sensitivity 100% Specificity 82% PPV 0.380 NPV 1.00
Dahlgren et al. 1989 [42]	1579 (170 EC cases)						
Baak et al. 1988 [43]	2652 (480 EC cases)						

*Abbreviations:* *AUC* area under the receiver-operator curve, *E/O* ratio expected to observed ratio, *PPV* positive predictive value, *NPV* negative predictive value, *SD* standard deviation

Five models included measures of calibration: Shi et al. [28] tested their epidemiological model in the NHS, NHS II, and PLCO cohorts. They reported poor calibration in the NHS, with an expected-to-observed ratio of 0.55 (95% CI 0.51–0.59, suggesting a lack of fit. The epidemiological model was better calibrated in the NHS II cohort, with an expected-to-observed ratio of 1.09 (95% CI 0.98–1.22). The expected-to-observed ratio of 1.04 (95% CI 0.95–1.13) in the PLCO cohort suggested that the epidemiological model was well-calibrated, with a slight overestimation of outcomes. For the epidemiological and genetic model, measures of calibration were only provided from the PLCO cohort, with the expected-to-observed ratio of 0.94 (95% CI 0.85–1.03) suggesting a slight under-prediction.

Hüsing et al. [36] reported a ratio of expected-to-observed cases of 0.99, indicating good calibration. Pfeiffer et al. [39] also provided a ratio of expected-to-observed cases of 1.20 (95% CI 1.11–1.29), suggesting their model tends to overpredict. For both of their models, Hippisley-Cox and Coupland [37, 38] provided calibration plots of mean predicted risks and observed risks at 10 years by tenths of predicted risk. In both papers they interpreted these plots as showing the models to be well calibrated.

### Performance of models developed in high-risk populations

Nine models were designed to predict EC in a high-risk population [25–27, 30, 32, 34, 40, 42, 43]. Three had a high AUC: Ruan et al. [27], Giannella et al. [34] and Bagepalli Srinivas et al. [32], and Ruan et al. [27] externally validated their model by testing it in patients from a different time period from the development patients from their dataset, reporting AUCs of 0.91 (95% CI 0.88–0.94) in external validation and 0.84 (95% CI 0.80–0.87) in development. Giannella et al. [34] had an AUC of 0.85 (95% CI 0.80–0.90) and Bagepalli Srinivas et al. [32] had an AUC of 0.85 (95% CI 0.75–0.93). Three studies reported moderate AUC, with AUC for different miRNAs ranging from 0.78 (95% CI 0.66–0.91) to 0.62 (95% CI 0.48–0.77) in Lin et al. [30]. Rewcastle et al. [26] reported an AUC of 0.77 (95% CI 0.66–0.79), whilst Burbos et al. [40] reported an AUC of 0.77.

Of the models designed for high-risk populations, only one, Ruan et al. [27], provided a measure of calibration. They included plots of predicted and observed frequencies for both development and validation cohorts, with mean absolute errors of 0.005 and 0.053 respectively, interpreting these as showing ‘good’ calibration in development and ‘acceptable’ calibration in validation.

## Discussion

In this review, we have identified and analysed 20 papers [24–43] on 19 EC risk prediction models. Most models had a moderate (AUC 0.60–0.80) or high discriminative ability (AUC > 0.80). Analysis of the risk of bias using the PROBAST tool found three models to be rated as low risk. All three were designed for the general population. All models included in the study had low concern regarding applicability as their participants, predictors, and outcomes were appropriate for the review question. Of the models with an overall low risk of bias, the Hippisley-Cox and Coupland QCancer models had the best performance, with high AUCs of 0.83 and 0.91 and good calibration [37, 38]. Hüsing et al. [36] reported a moderate AUC of 0.77 for their model, which was also well-calibrated with an expected-to-observed ratio of 0.99.

The most commonly used method of model development was logistic regression. This may be due to its appropriateness given the study designs used, simplicity, and interpretability; however, this binary approach may fail to fully capture the temporal aspect of EC, as we know cancers develop slowly, over years. Perhaps time-to-event models such as Cox regression (which was used by four models) in prospective studies could offer a better understanding of EC risk, especially when considering the time until an event occurs. Efforts should be made to compare these types of model development in an EC risk prediction setting to determine differences in their performance. These models could also be compared with more novel approaches, including machine learning methods, to guide future model development.

One of the main issues regarding EC diagnosis is the high sensitivity but low specificity of abnormal bleeding for EC [10]. According to the 2015 NICE guidelines, last updated in February 2021 [44], any woman over 55 with postmenopausal bleeding is referred via the 2-week-wait pathway for suspected cancer and is offered an endometrial biopsy.

Models designed for the primary care setting like those described by Hippisley-Cox and Coupland [37, 38] are therefore perhaps the most applicable and useful, as they do not require endometrial tissue and demonstrate good discrimination based on symptoms easily identified in primary care without the need for invasive testing. These models are developed using electronic records from primary care settings, which can be heterogeneous between practices due to factors such as different practice policies and coding systems. As a result, model variables may reflect symptoms and diagnoses that are commonly recorded by the practices included. Whilst the Hippisley-Cox and Coupland models perform well in external validation in a different UK cohort [37, 38], validation in a non-European cohort may demonstrate the utility of these models in different populations.

### Risk of bias

The PROBAST tool [23] provides firm conditions for the ratings of risk of bias and applicability of models. In particular, if a model receives a rating of high risk of bias in a single domain, the overall rating must be high risk. Fourteen models were assigned a high or unclear risk of bias under the analysis domain [24–27, 29–35, 40, 42, 43]. This was common because they did not report on one or both of discrimination and calibration, their ratio of model variables to EC cases was less than 10 in the development papers (or they included fewer than 100 EC cases in the validation cohorts), or they handled continuous variables inappropriately. Studies were also commonly penalised in the participants’ domain, often due to having a case–control design, with 12 studies receiving a rating of high or unclear risk of bias in this domain. Most models received an unclear risk of bias rating for outcomes, due to failing to specify their time frame of risk prediction.

In PROBAST, applicability refers to how relevant to the review question the participants, predictors, and outcomes of the models are. All models included were deemed to have low concern regarding applicability, which may reflect the broad scope of the review question.

PROBAST does not explicitly consider model generalisability. Two of the models, Shi et al. [28] and Pfeiffer et al. [39] were designed specifically for white women. These models reported moderate-low discrimination, but this may be lower if the models were to be applied to women of all ethnicities. An essential area for future development of EC risk models is validation in diverse cohorts including different ethnic backgrounds, to assess whether models can be used in different populations. It has been shown that women of different ethnicities have different risks of EC [45]. This could highlight a need for population-specific model adaptations, as, for example, factors affecting the likelihood of EC development such as parity, HRT use, combined oral contraceptive pill use, and obesity vary largely between countries and cultures.

### High-risk populations

High-risk populations were defined as including women with abnormal uterine bleeding [27, 32, 34, 40], endometrial hyperplasia [26, 30, 41, 43], or those who had been referred to gynaecological oncology [42]. In order to define women as high-risk on the basis of having endometrial hyperplasia, they would have had to first undergo an ultrasound scan. These are typically only offered to women presenting with abnormal bleeding; hence, papers defining women as high-risk based on endometrial hyperplasia are likely to sample only a small proportion of women, those with concurrent symptoms. This method of recruitment is used in Bagepalli Srinivas et al. [32] and Giannella et al. [34] meaning they possibly both are susceptible to such sampling bias. Of the nine models developed in high-risk populations, all had a high risk of bias in the ‘analysis’ domain. Three reported high AUCs, 0.91 for Ruan et al. [27], 0.85 for Giannella et al. [34], and 0.85 Bagepalli Srinivas et al. [32]. Giannella et al. and Bagepalli Srinivas et al. were intended for use in premenopausal women. Giannella et al. included endometrial thickness of greater than 11 mm in the model. The utility of endometrial thickness as a risk factor in premenopausal women is unclear, as endometrial thickness can exceed 11 mm during the luteal phase of the menstrual cycle [46]; therefore, external validation of this model would be important to assess its performance.

Overall, the QCancer models [37, 38] emerged as the most promising models, because they demonstrated a high AUC, good calibration, and low risk of bias, and are likely to be the most applicable due to their targeted use in primary care. Additionally, both papers use large development and validation cohorts. The other models with high AUC typically required more invasive testing or were ranked with a high risk of bias. Both of the QCancer models [37, 38] predict risk over a short-term period (2 and 5 years) as opposed to lifetime risk, hence perhaps may be more useful in informing care decisions rather than in motivating lifestyle changes.

Interestingly, many models were missing established risk factors. Only two models included a family history of cancer [27, 37], and no models included genetic syndromes associated with EC, such as Lynch syndrome, which underlies 3% of EC cases [19]. Further, women with Lynch syndrome often undergo preventative surgery such as hysterectomy with salpingo-oophorectomy. Development of risk models for this group of women could help with decision-making regarding these procedures and their optimum timings to delay early menopause and prolong childbearing potential, whilst avoiding the development of EC. It has also been suggested that BRCA1 may be associated with EC [47]; however, this may be due to its strong association with breast cancer and hence tamoxifen use. Of the papers screened for eligibility, tamoxifen use was only included in a single model, Kitson et al. [48], which consists of a total score calculated by combining obesity, reproductive, insulin, and genetic sub-scores. This model is currently entirely theoretical and has not been tested or validated.

All but four papers (Shi et al. 2023 [28], Hüsing et al. 2016 [36], and both of the Hippisley-Cox and Coupland papers [37, 38]) did not provide a time frame over which they were predicting risk. This brings into question the potential for utilisation of these models, as it is more difficult to discern the time frame over which they are predicting risk. In fact, the studies were conducted over time frames ranging from 3 years [40] to up to 19 years [39].

Assessment of model quality is essential and PROBAST is an expert-informed and rigorously developed tool designed for this purpose. However, for a model to be suitable for use in practice, external validation in the target population is highly desirable which is only one of several aspects considered in PROBAST.

Whilst there exists a prior systematic review of EC risk prediction models, Alblas et al. [49], we believe that our review has captured a different subset of models and is the first to focus specifically on models with an application in the prediction of future risk of cancer development, as opposed to diagnostic models. Eleven of the models we include were published after their study, and they also omit both of the Hippisley-Cox and Coupland papers representing the QCancer models [37, 38], which we deemed appropriate for inclusion, a fact which the authors of these models commented on [50]. Indeed, both of these papers had a high AUC and were developed and validated in large cohorts, representing the most promising models included. Therefore, we believe our paper represents an accurate and updated review of models predicting future EC risk.

### Implications

It is important to question whether there is a need to predict the risk of EC. Whilst EC is the most commonly diagnosed gynaecological cancer in some populations, it often presents early in primary care with abnormal bleeding and can be referred for further investigation. There is no screening test for EC nor has there even been a demand for one [51]. A screening programme would only be feasible if there was a rapid, low-cost test with very high specificity to account for the fact that EC is relatively rare. Symptoms of EC typically present early in the disease; given the purpose of screening is to identify early disease it seems there is little benefit in screening for EC.

Risk prediction models could help to overcome key gaps in diagnosis and treatment of EC such as risk scoring and grouping of women into risk stratification groups. Further, risk scoring could help to stratify women into those requiring more drastic investigation and those in whom a more minimally invasive approach would suffice. There are key subgroups who could benefit by the use of risk prediction models and who warrant further investigation, for example, women at risk of more aggressive types of EC and those genetically predisposed.

As for any disease where lifestyle factors may play a role in its development, risk models could play a vital role in the identification of at-risk women whose risk could be optimised by lifestyle interventions. This approach to risk reduction has been proven before; intentional weight loss has been shown to reduce EC risk [52]. Perhaps medications such as the combined oral contraceptive pill may play a role in long-term risk prevention, acting to regulate oestrogen levels and hence reduce EC risk. It is already used for this purpose in the management of PCOS. A potential further feature of risk prediction models could be to estimate the decrease in risk associated with lifestyle interventions, which could inform women on the benefits of these before embarking on them. Where risk models are being used to identify candidates who may benefit from lifestyle interventions, models which predict over a longer time frame of many years may be more appropriate and pragmatic, given implementing lifestyle changes is often a slow process and any resulting changes in risk would be expected to happen gradually.

It is important to consider the clinical impact of the models and how, if at all, they may change clinical practice. In hand with this, the study of this impact is essential. The QCancer models are available for use in UK general practices; however, a study by Price et al. [53] assessing its implementation found that it is underutilised. Qualitative studies have identified potential barriers to the use of QCancer including consultation time, potential for over-referral, unnecessary worry of patients, and a need for evidence for effectiveness [54]. This emphasises the need for external validation of risk prediction models before their clinical implementation and highlights barriers which are applicable to all risk prediction models designed for this setting. The other models described in this review are yet to be implemented in a clinical setting, with a lack of external validation potentially a key factor in this. Other points to consider in real-world model implementation include the ease of application, which in turn is influenced by the variables included in the model. Practically, models requiring data from laboratory assays would be much more difficult to implement in a wider setting and perhaps would be more appropriate for selective use in a high-risk population. Assessment of clinical outcomes will be vital in determining how important risk prediction modelling of EC is. Future research should focus on prospective studies or trials to quantify the real-world impact of these models, looking at factors such as earlier diagnosis of EC and survival rates.

## Conclusion

Several models exist which could be used to predict the risk of short- and long-term risk of EC, with good predictive performance. However, it is important to validate models in larger and more diverse cohorts. Once their applicability is established, they could be explored as aids in making clinical judgement and informing lifestyle choices with the aim of preventing EC.

## Supplementary Information

Additional file 1: eFigure 1 PRISMA flowchart of the systematic literature search conducted for gynaecological cancers risk prediction models.

Additional file 2: Appendix S1

## Data Availability

The dataset supporting the conclusions of this article is included within the article (and its additional files.

## Abbreviations

EC: Endometrial cancer
PMB: Postmenopausal bleeding
DM: Diabetes mellitus
PCOS: Polycystic ovarian syndrome
AUROC: Area under the receiver operating characteristic curve
PPV: Positive predictive value
NPV: Negative predictive value
