# Supplementary material for: Models for predicting risk of endometrial cancer: a systematic review
Source: Diagn Progn Res. 2025 Feb 4;9:3. doi: 10.1186/s41512-024-00178-0 (PMC11792366; doi:10.1186/s41512-024-00178-0)
Supplement: Supplementary file 1 — Additional file 1: eFigure 1 PRISMA flowchart of the systematic literature search conducted for gynaecological cancers risk prediction models. [file 41512_2024_178_MOESM1_ESM.docx]

eFigure 1. PRISMA flowchart of the systematic literature search conducted for gynaecological cancers risk prediction models.

Records removed *before screening*:

Duplicate records removed

(n = 4,213)

Records identified from Medline and Embase to December 3rd, 2023

n = 10,707

**Identification**

Records screened

(n = 6,494)

Records excluded on abstract and title

(n = 6,371)

**Screening**

Studies on gynaecological cancers other than ovarian excluded:

ovarian (n = 42)

cervical (n= 20)

Full-text articles assessed for eligibility

(n = 123)

Reports excluded:

different outcome

irrelevant design (n = 2)

diagnostic tool (n = 3)

**Eligibility**

Full-text articles on endometrial/uterine cancer

(n = 23)

Reports added from references

(n = 2)

Studies included in the review

(n = 20)

**Included**

eTable 1. List of participating countries in the European Prospective Investigation into Cancer and Nutrition (EPIC) and FORECEE cohorts in the included endometrial cancer (EC) risk prediction models.

| Cohort name | Participating countries |
| --- | --- |
| EPIC Cohort | Norway, Sweden, Denmark, the United Kingdom, the Netherlands, Germany, France, Spain, Italy, Greece |
| FORECEE | UK, Czech Republic, Italy, Norway, Germany |
